# Point-of-Care Diagnostic Testing for Emerging and Existing Poultry Viral Respiratory Pathogens Using Loop-Mediated Isothermal Amplification

**DOI:** 10.3390/pathogens14070657

**Published:** 2025-07-02

**Authors:** Ben Enyetornye, Aurelle Yondo, Binu T. Velayudhan

**Affiliations:** 1 Athens Veterinary Diagnostic Laboratory, College of Veterinary Medicine, University of Georgia, Athens, GA 30602, USA; 2 Department of Pathology, College of Veterinary Medicine, University of Georgia, Athens, GA 30602, USA

**Keywords:** LAMP/RT-LAMP assay, poultry respiratory pathogens, point-of-care testing, avian influenza, Newcastle disease, infectious bronchitis, infectious laryngotracheitis, avian metapneumovirus

## Abstract

Accurate, rapid and inexpensive diagnosis of poultry respiratory pathogens remains a challenge, especially in many developing countries. Meanwhile, poultry respiratory pathogens are a major threat to poultry production worldwide, accounting for billions of dollars in economic loss to the sector. Early and accurate diagnosis of these diseases is critical for economic poultry production. Molecular diagnostic methods, including PCR-based techniques, have been developed and used to fill this gap, but unfortunately, these techniques require skilled technicians, relatively costly equipment and reagents and can only be performed in a laboratory setting. This warrants the development of other diagnostic tools, which can be used in the field even by unskilled personnel. In this review, we discussed the genesis, challenges, advances and prospects of loop-mediated isothermal amplification (LAMP) for the detection of poultry respiratory pathogens at the flock side, especially in resource-constrained countries. We highlighted the application of LAMP in routine poultry disease surveillance and early outbreak detection, underscoring its value as a transformative diagnostic tool in poultry production. The development and use of a point-of-care (POC) LAMP assay that can be used to screen for these poultry respiratory pathogens simultaneously enhance disease surveillance and diagnosis.

## 1. Introduction

Poultry respiratory viral pathogens, including infectious bronchitis virus (IBV), infectious laryngotracheitis virus (ILTV), Newcastle disease virus (NDV), avian metapneumovirus (aMPV) and avian influenza virus (AIV), remain a global threat to poultry production [1], especially in resource-constrained countries where veterinary diagnostic laboratories are scarce, and there are limited funds to purchase sophisticated equipment, supplies and relevant reagents for the diagnosis of disease and for disease surveillance [2]. In places where farmers or field veterinarians have access to a veterinary diagnostic laboratory, the cost of laboratory diagnosis is often very expensive, and/or the turnaround time of laboratory results is longer, making it difficult to initiate appropriate interventions in a timely manner during disease outbreaks. In the laboratories, relatively expensive instruments and skilled professionals are required to conduct screening of samples using molecular techniques such as RT-PCR [3,4]. These challenges keep field poultry veterinarians in a fix, making them rely largely on clinical signs, disease history and experience for diagnosis of poultry respiratory diseases [5]. This approach could be troublesome, especially because most of these poultry respiratory diseases present non-specific and similar clinical signs, impeding a precise clinical diagnosis in the absence of laboratory testing [3]. There can also be co-infections of poultry with these viruses followed by secondary bacterial infections; hence, relying on clinical signs and disease history alone for diagnosis is not recommended.

Meanwhile, poultry respiratory pathogens continue to spread rapidly in many poultry production systems all over the world, with avian influenza currently dominating the global health landscape, posing a public health risk of unprecedented magnitude [6]. Newcastle disease virus, another important poultry pathogen, has been identified to cause a transboundary spread across eastern, western, central and southern Africa [7], causing significant outbreaks in southeast Asia, particularly in Indonesia in recent years [8]. The virulent strains of Newcastle disease virus are present in Mexico [9], posing a great threat to commercial poultry in neighboring countries like the United States of America and Canada, although wild birds have been found to harbor the virus in both countries [10,11]. The increasing cases of aMPV is an intercontinental problem. In Europe, since the introduction of aMPV in 1985 in France, the virus continued to spread through the Mediterranean and Western Europe [12]. In 2024, aMPV subtype A was reported in a turkey flock in California in the USA. This aMPV strain was phylogenetically related to the aMPV reported in Mexico in 2022, suggesting a transborder spillover between these two countries. These viral pathogens are currently distributed globally, posing a great threat to poultry across the world [13,14,15,16,17].

Considering the rapid spread and global distribution of these viral pathogens, an inexpensive, rapid and specific point-of-care test is ideal to enable poultry veterinarians, farmers and breeders to implement timely and cost-effective measures, with the goal of quickly containing these viral agents [18]. A promising point-of-care test, which is increasingly gaining recognition in the diagnosis of poultry diseases, especially those affecting the respiratory system of chickens, is loop-mediated isothermal amplification (LAMP) [19]. The LAMP technology is a fast, robust and reliable diagnostic platform for point-of-care testing [20]. Recent developments of various methods for the sequence-specific detection of LAMP show high potential for many future applications [21]. Several techniques, such as recombinase-aided amplification using isothermal amplification, have recently been proposed [22].

## 2. The Genesis of Loop-Mediated Isothermal Amplification

Traditionally, many viral pathogens have been diagnosed through selective isolation followed by electron microscopy. However, a major setback to this approach is the delay in the implementation of treatment and/or control measures in both developed and resource-constrained countries [23]. This challenge has led to an increased focus on the amplification of nucleic acid, which has become an invaluable tool in the diagnosis of infectious diseases present in both humans and animals [24,25]. For instance, many PCR-based techniques have been developed with the goal of enhancing disease diagnosis [26]. These PCR techniques have been widely used with great success. However, these techniques require high-precision instruments for amplification or an elaborate method for detection of amplified products [27]. The use of multiple primers in nested PCR has improved amplification specificity for the target sequences [28], but there is still residual co-amplification of unwanted sequences, making it a critical setback. A major requirement for PCR is a high-precision thermal cycler with a continuous power supply, making it a great limitation for use in resource-constrained laboratories or countries [29]. This situation warrants the development of a highly sensitive and specific diagnostic tool, which could amplify very few copies of DNA under isothermal conditions—the reason for which the LAMP technique was developed in the year 2000 [30]. The major characteristics of this LAMP assay are rapidity under isothermal conditions, high specificity for the target sequence and low reaction temperature (63–65 °C) requirement. To run a LAMP assay, four to six primers, a DNA polymerase and a regular laboratory water bath or heat block are required for reaction, making it easy to perform [30,31]. Since large amounts of amplified DNA are obtained from the LAMP reaction, this allows for effective detection of viral DNA with the naked eye due to color change or turbidity, depending on the levels of magnesium ions during reactions [3,32].

## 3. The Basic Principle and Primers Used in the LAMP Assay

The underlying basic principle of LAMP is the auto-cyclic strand-displacement reaction, which occurs at a constant temperature in the presence of a DNA polymerase. When a double-stranded DNA is in a dynamic equilibrium at a constant temperature, one of the LAMP primers can anneal to the complimentary sequence of the double-stranded DNA target, initiating DNA synthesis using the DNA polymerase, thereby displacing and releasing a single-stranded DNA [30,33]. A critical stage in the development and operationalization of the LAMP assay is the primer design stage. The primers ought to be optimized with regard to factors such as primer concentration, the location of nucleotide pairs and the distance between DNA regions [34]. The set of six primers used in the design of the LAMP assay are the forward internal primer (FIP) and the backward internal primer (BIP), which are collectively called the internal primers; the external primers (forward primer (F3) and backward primer (B3)); and the optional loop primers (loop primer forward (FL) and loop primer backward (BL)) [34,35]. The internal primers are about 45–49 bp long and complementary to the sense and antisense strand on the template, while the external primers are relatively shorter (21–24 bp), and they bind with the template more slowly, as they are applied in lower concentrations in the reaction mixture [34]. The internal and external primers, combined with the Bst DNA polymerase, which exhibits a high strand-displacement activity at 60–65 °C, create a dumbbell-like DNA structure, which serves as a template for further amplification [36].

## 4. Advantages of the LAMP Assay

**Figure 1:**
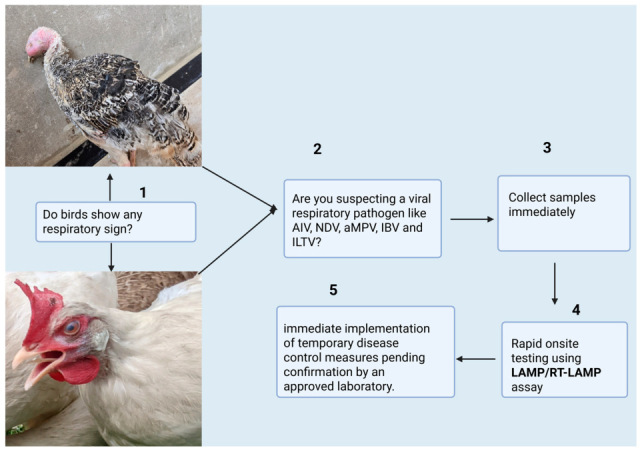
Illustration of the workflow using the LAMP/RT-LAMP assay in the diagnosis of poultry respiratory viruses.

A major advantage of LAMP is its rapidity, as it allows immediate diagnosis (Figure 1) in comparison with other nucleic acid amplification techniques, such as PCR. LAMP can be performed in about 30 min, whereas PCR requires at least 90 min (Table 1). LAMP amplification is rapid because the initial heat denaturation for the DNA template is not required [37]. The enzyme Bst DNA polymerase, which is isolated from *Geobacillus (Bacillus) stearothermophilus*, plays an important role in LAMP reactions [38]. This enzyme has strand-displacement activity, releasing single-stranded DNA during synthesis, thereby bypassing the denaturation stage [30]. Moreover, the LAMP assay can be used directly on clinical or non-processed samples, thereby bypassing the extraction step, which must be performed for other nucleic acid detection systems like PCR [39,40]. This is because LAMP exhibits reduced sensitivity to inhibitor substances in biological samples compared to PCR [41]. Additionally, the development and use of pH-sensitive dye in LAMP reactions make it easy to visualize color [42] due to the reaction, making it a promising diagnostic tool for use on site in resource-constrained countries. When nucleic acid is amplified by LAMP, the turbidity generated by the precipitate is based on the progress of the reaction, which enables easy monitoring with the naked eye [35]. Another feature of LAMP is that it is simple, cost-effective and practical for laboratories that are under-equipped or for field conditions, since all that is required is a water bath or heating block that serves as a source for isothermal conditions [27,37]. Also, the LAMP assay has high specificity because there is improved amplification reaction when all six regions within a target DNA are correctly recognized by the primers. The addition of reverse transcriptase enables the amplification of DNA from RNA sequences with high efficiency [23]. Hence, LAMP is used for DNA viruses, while RT-LAMP can be used for RNA viruses. An excellent feature of LAMP is its ability to produce high volumes of white magnesium pyrophosphate precipitate in positive reactions [31], enabling easy visual identification of positive reactions [31,43] and avoiding the additional cost and labor entailed by post-amplification analysis [44]. The closed-tube LAMP method can minimize the problem of contamination in less controlled environments [44]. The LAMP technique allows for the development of POC diagnostic platforms, which can be extremely useful in resource-constrained countries, as they are small, portable, user-friendly and affordable [45].

## 5. Challenges with Developing LAMP Assays

The LAMP assay remains a very important diagnostic tool for the detection of many poultry pathogens [46]. However, there are several drawbacks that must be addressed to ensure its broader application and reliability in field, clinical and laboratory settings. The success of LAMP depends largely on proper primer design. This requires an in-depth knowledge of molecular biology [47]. LAMP uses four to six primers to specifically amplify six to eight regions of the target DNA [30,48]. Designing these primers is a complex process that requires careful consideration of several key factors, including the melting temperature, GC content, primer length, ΔG thresholds and the spacing between priming sites [49]. Although LAMP primer design tools are available, sometimes, the software fails to include loop primers in the generated design output, necessitating manual adjustments [50]. Non-specific amplifications are commonly observed in LAMP reactions, largely due to the number of primers required and the challenge of designing them optimally [51]. This increases the risk of secondary structure formation, potentially impacting the reliability of the assays [52]. In some cases, redesigning primers is necessary, making it a more laborious and time-consuming process. Moreover, designing primer sets to detect novel pathogens requires extensive knowledge of the pathogen’s genome, which may not always be readily available. Additionally, if the target region has high sequence variability, the primers must be designed to target the most conserved regions to ensure that amplification is not hindered by genetic variation in the target gene of interest.

Another limitation of LAMP is the difficulty in detecting multiple targets simultaneously. PCR-based methods can easily accommodate the detection of several targets in a single reaction by using different sets of primers. However, due to the complex primer requirements in LAMP, multiplexing becomes more challenging and increases the likelihood of non-specific amplification [53]. As a result, developing multiplex LAMP assays that can reliably distinguish between different targets remains an area to be effectively explored, although there have been some advances in recent times.

LAMP is well known for its high sensitivity, enabling the detection of very small amounts of DNA. However, the assay is also highly susceptible to contamination, which can lead to false positives [54]. To prevent contamination and misleading results, strict laboratory protocols and careful handling of reagents are essential. While the LAMP assay is well suited for low-resource settings, maintaining high standards of sterility in such environments can be challenging due to the increased risk of environmental contaminants.

LAMP operates most efficiently between 60 and 69 °C under isothermal conditions, with an optimal temperature of 65 °C, in contrast to PCR, which uses multiple temperature cycles for amplification [4,55]. Determining the optimal temperature, primer and magnesium concentrations can be a drawback of using LAMP assays, as variations in these conditions may heavily affect the amplification process and lead to inconsistent results. For instance, suboptimal temperatures can result in incomplete or reduced amplification, impairing the assay’s ability to detect target DNA or RNA at low concentrations.

While LAMP is highly effective in controlled laboratory conditions, sometimes, challenges may arise when it is applied to clinical samples, such as blood, tissue or saliva. These samples may have low viral loads and often contain inhibitors that can affect the reaction’s efficiency, requiring robust amplification. Pre-treatment of samples to remove inhibitors can complicate the workflow and reduce the simplicity and rapidity, which make LAMP attractive for POC applications. Furthermore, biosafety concerns have been raised during the validation process regarding direct sample handling to avoid exposure to live viruses [56].

LAMP results are often detected using colorimetric or fluorescence-based methods [57,58]. Colorimetric detection is simple and cost-effective. However, the visual interpretation is subjective and may lead to false positives or negatives, especially in low-resource settings. Fluorescence-based detection offers more precision but requires specialized equipment, which may not be available under such conditions.

LAMP is primarily a qualitative assay, meaning it indicates whether the target nucleic acid is present or absent, but it does not provide quantification. Meanwhile, managing certain viral infections requires viral load monitoring [59]. Therefore, quantitative analysis, such as real-time PCR (qPCR), remains critical in such situations.

The current LAMP reaction workflows often involve manual sample preparation, reaction setup and detection, which can be time-consuming and error-prone. There is a need for better automation systems that would be ideal for field applications, particularly in high-throughput conditions. Developing automated systems for LAMP would significantly improve its utility for larger-scale testing, such as in poultry farms, hospital laboratories or during disease outbreaks.

## 6. Detection of Poultry Viral Respiratory Pathogens Using LAMP

To date, a number of LAMP assays have been developed to enhance the diagnosis of major viral respiratory pathogens (ILTV, INV, aMPV, NDV and AIV). There are currently ongoing studies to improve upon the existing LAMP assays. Some advances made in the development of LAMP assays for the diagnosis of common viral respiratory pathogens are summarized below.

### 6.1. Iltovirus gallidalpha1 (Formerly Called Infectious Laryngotracheitis Virus)

*Iltovirus gallidalpha1*, originally called ILTV, is a contagious upper respiratory tract disease in chickens caused by a Gallid herpesvirus 1 (GaHV-1), belonging to the genus *Iltovirus* and subfamily *Alphaherpesvirinae* within family *Orthoherpesviridae* [60]. The disease is characterized by conjunctivitis, sinusitis, oculo-nasal discharge, respiratory distress, bloody mucus, swollen orbital sinuses, high morbidity, considerable mortality and decreased egg production [61]. Traditionally, the diagnosis of ILT has required laboratory assistance, as making diagnosis based solely on clinical disease presentation can be misleading, given that other poultry pathogens present similar clinical signs and lesions [62]. To enhance control, rapid diagnosis is key [62], making LAMP a suitable diagnostic tool, especially for field conditions.

In southern China, the ILTV-LAMP assay has been shown to be a great alternative diagnostic method for diagnosis of ILTV infections in primary care facilities and poorly equipped laboratories. This method demonstrated about ten times greater sensitivity than PCR and was highly specific, as there was no cross-reaction with other avian viruses. This LAMP assay has been shown as an effective method for the detection of ILTV, with the entire assay duration being 40 min. LAMP primers were designed to target the TK protein gene, and LAMP reaction was carried out in a conventional water bath. LAMP amplicons were visualized with the naked eye after the addition of SYBR Green I [63]. Later, Ou et al. [64] also developed a LAMP assay to detect Gallid herpesvirus 1. Although the sensitivity of this LAMP assay was lower than that of real-time PCR, this LAMP was faster, less expensive and did not require the use of a thermocycler. In this assay, the primers were designed to target the ICP4 gene of Gallid herpesvirus 1, amplifying the target gene at 65 °C within 45 min, and the limit of detection of the assay was 60 copies/μL.

Quite recently, a modified LAMP assay was developed in which the cap remained closed after the amplification reaction. The goal was to avoid contamination, thereby preventing the chances of false-positive results. In this assay, the LAMP reaction occurred at 66 °C, and the turbidity of the LAMP reaction was measured using a real-time turbidity meter, with a minimum detection concentration of 0.06 pg/lL (equivalent to 353 copies/lL), making it 100-fold higher than that of PCR. Generally, electrophoresis or SYBR Green I fluorescent dye is used for the detection of LAMP reactions. However, both of these methods expose the reaction products to the air, thereby contaminating the environment, and they are also extremely prone to false positives. A major modification in this new LAMP assay was the integration of a real-time turbidity meter and Calcein to detect the LAMP reaction. The researchers opined that this newly designed assay might be a reliable method for facilitating the surveillance and clinical diagnosis of ILTV [65]. El-Tholoth et al. [18] later developed a rapid, simple, semi-quantitative benchtop LAMP assay and a field-deployable microfluidic device for the diagnosis of ILTV infection in chickens. The sensitivity of the LAMP assay was 250 genomic copies/reaction. This assay was proposed by the researchers as suitable for field surveillance of ILT, especially in low-resource veterinary diagnostic laboratories.

### 6.2. Infectious Bronchitis Virus

Infectious bronchitis virus is a highly contagious avian *Gammacoronavirus* (*Gammacoronavirus galli*) that mainly affects chickens (*Gallus gallus*) but can circulate in other avian species. This virus belongs to the subfamily *Orthocoronavirinae* and family *Coronaviridae* [66]. IBV poses a significant threat to the global poultry industry, causing reduced egg production and growth, with an increased mortality rate [67]. The surveillance and identification of IBV types [68] are critical to the control of viral infection. However, clinical diagnosis is challenging, as clinical and post-mortem findings are not pathognomonic [69]. The development and use of LAMP assays that can effectively detect IBV fit well with the global surveillance goal.

In 2010, an RT-LAMP assay was developed targeting the nucleocapsid phosphoprotein gene of IBV. The limit of detection of this assay was 10^1^ EID50 per 50 mL of titrated virus. There was no cross-reaction of this IBV RT-LAMP assay with other poultry viruses, such as NDV, reovirus and ILTV. A perfect correlation existed between all clinical samples tested using RT-PCR and this RT-LAMP assay. Comparatively, the sensitivities of this IBV RT-LAMP assay and RT-PCR were 99.5% and 98.4%, respectively, with both assays exhibiting 100% detection for blood samples. This suggests that this RT-LAMP can be used not only for disease diagnosis but also for poultry disease surveillance. Diagnostic results could be obtained in just 45 min using this IBV RT-LAMP assay [70]. Another research group also developed a LAMP assay for rapid diagnosis of IBV, with primers targeting the spike protein 2 gene (S2). Compared with RT-PCR, this LAMP assay was 100 times more sensitive, with a specificity of 94% for the S2 gene. The RT-LAMP primers used were optimized to run at 60 °C for 45 min [71]. Later, RT-LAMP was combined with lateral-flow dipstick, forming a novel detection tool, which was capable of simultaneously detecting IBV and NDV. The RT-LAMP primers were designed to target the 5′-untranslated region (5′-UTR) of the IBV genome and the conserved region of the NDV large polymerase gene (LP). With regard to the sensitivity of this assay for IBV or NDV alone, the lowest detection limits were 10^0.8^ IBV RNA copies/reaction and 10^0.7^ NDV RNA copies/reaction, respectively. The same detection limits were recorded when this novel RT-LAMP assay was used to detect IBV and NDV simultaneously. This assay served as a promising detection tool suitable for field detection of IBV and NDV co-infections [72]. In 2020, a rapid, simple, semi-quantitative, closed-tube, single-step, real-time RT-LAMP assay was developed with the goal of reducing contamination to reduce the risk of false positives. This assay showed great concordance with RT-qPCR when used for testing of clinical samples, with a limit of detection of 1 EID_50_/mL [73].

### 6.3. Avian Metapneumovirus

Avian metapneumovirus is a highly contagious pathogen, which infects turkeys and chickens, resulting in turkey rhinotracheitis (TRT) and swollen head syndrome (SHS), respectively [74]. The causative agent is avian metapneumovirus, which belongs to the family *Pneumoviridae* [75]. Quite recently, a novel, rapid and affordable RT-LAMP assay has been developed for the diagnosis of aMPV. Its detection limit was comparable to RT-qPCR, with a completion time of 60 min, and it was suitable for field use, with 100% specificity and 87.88% sensitivity. Oligonucleotide probes were designed to target a conserved 17 nt long sequence in the viral F gene [3]. There seem to be limited studies designing RT-LAMP assays for field detection of avian metapneumovirus.

### 6.4. Newcastle Disease Virus

Newcastle disease is an acute and highly contagious disease of poultry caused by *Orthoavulavirus javaense*, belonging to the subfamily *Avulavirinae* and family *Paramyxoviridae*. This disease has caused great harm to the poultry industry globally. Rapid diagnosis of ND is key to early symptomatic treatment and implementation of control measures [76]. To support this goal, a LAMP assay was developed in 2005 to enhance rapid, simple and sensitive detection of NDV directly from both clinical samples and culture isolates. LAMP primers were designed to target the fusion protein gene. In comparison with nested PCR, this LAMP assay was as specific and sensitive but faster, cost-effective and easy to carry out [77]. However, this LAMP assay was reported to produce serious false-negative results under field conditions, warranting the need to develop a modified LAMP assay by other researchers [27]. In the development of this assay, six RT-LAMP primers with degenerate oligonucleotides were designed to target a highly conserved region of the fusion gene. This improved RT-LAMP assay detected 21 NDV isolates, with no cross-reaction with other avian viruses, and it could be performed in about 50 min. Also, this assay was five times more sensitive than the previously developed LAMP assay, achieving 96.8% sensitivity with a total of 62 samples (30 field clinical samples, 24 experimentally infected samples and 8 experimentally negative samples). This improved RT-LAMP assay provided a simple, rapid and cost-effective method that was practical for use in under-equipped laboratories and in the field [27]. Later in 2016, a rapid NDV detection system was developed by combining the high amplification efficiency of LAMP with an optomagnetic nanoparticle-based readout. This combination produced an ultrasensitive LAMP assay, which was capable of performing a quantitative readout; it was user-friendly and at a reduced cost. In this assay, biotinylated amplicons of LAMP and reverse-transcription LAMP were bound to streptavidin-coated magnetic nanoparticles (MNPs), resulting in a dramatic increase in the hydrodynamic size of the MNPs. This increase was measured by an optomagnetic readout system and provided quantitative information on the amount of LAMP target sequence. This assay had a limit of detection of 10 aM of the target sequence, with a total assay time of 30 min. The assay was proven to be specific and sensitive, as it produced comparable results with RT-PCR when used in the screening of clinical samples. Obviously, this assay may not only benefit epizootic surveillance of NDV but could also be adapted to detect other RNA viruses [78]. Other researchers also designed a multiplex LAMP and lateral-flow dipstick capable of simultaneously detecting NDV and IBV, as discussed in the IBV section [72].

### 6.5. Avian Influenza

Avian influenza is a contagious disease among poultry characterized by high mortality and decreased production, accounting for significant economic losses and exorbitant costs in terms of disease control and outbreak management. Avian influenza is caused by an *alphainfluenzavirus* belonging to the family *Orthomyxoviridae*, with only the influenza A virus being capable of infecting birds [79]. These avian strains are pathogenic to humans [80]. Over the years, there has been a proliferation of LAMP assays to detect various AIV subtypes. In one of these studies, a one-step reverse-transcription loop-mediated isothermal amplification assay for detection of highly pathogenic avian influenza (HPAI) H5N1 viruses was developed. This assay was tested with a panel of previously isolated HPAI H5N1 subtypes and clinical samples. The detection limit of this assay was found to be 2 × 10^−3^ plaque-forming units per reaction. In comparison with an optimized RT-PCR assay, their detection limits were identical when used in testing clinical samples. The results suggested that the sensitivities of these assays were comparable [44].

In another study, the most conserved region of the matrix gene sequence of influenza A was used for primer design to enhance the sensitivity and specificity of the RT-LAMP assay. A wide variety of samples from wild and domestic avian species originating from different geographical areas were tested to confirm the validity of these LAMP primers. This RT-LAMP assay was useful in detecting AIV in various field samples, such as swabs, tissues and feces, within 1 h [81]. Due to the relatively high predominance of H3 subtypes of low pathogenic avian influenza viruses, other researchers designed a LAMP assay based on primer sets targeting the sequences of the hemagglutinin (HA) gene of H3 subtype. This LAMP assay was used to test 176 clinical samples, of which 38 were H3 subtype, with these results being consistent with virus isolation. The assay could be performed in 50 min, with the amplification results visible with the naked eye and a detection limit of 0.1 pg total RNA of the virus, making it 100-fold higher than that of RT-PCR [82]. Due to the emergence of a novel reassortant avian influenza A (H7N9) in China in March 2013, which was rapidly spreading among humans, an RT-LAMP assay was developed to enhance rapid detection. The detection limit of this RT-LAMP assay was 0.01 PFU H7N9 virus, making this method 100-fold more sensitive than conventional RT-PCR in the detection of H7N9 influenza virus. This RT-LAMP assay could detect H7N9 influenza viruses from various sources (humans, pigeons, chickens and the environment). This RT-LAMP assay could effectively detect H7N9 influenza virus RNA in drinking water, soil, cloacal swab and tracheal swab samples that were collected from live poultry markets, as well as human H7N9 virus, in about 30 min, with no cross-reaction with other influenza subtypes or other poultry respiratory pathogens [83]. Around the same time, another research group also developed an RT-LAMP assay for detecting H7N9 influenza viruses. The sensitivity of this assay was 42.47 copies/reaction, with no cross-reactivity with seasonal influenza A (H3N2 and H1N1) or influenza B viruses in humans or with avian influenza A (H5N1) viruses. This RT-LAMP assay was highly specific and sensitive in detecting the avian influenza A (H7N9) virus, and the reaction was completed within 30 min [84].

For the rapid and visual detection of the M gene of all subtypes of avian influenza virus and the H5 gene of H5 subtype of highly pathogenic avian influenza virus (HPAIV), a two-tube reverse-transcription loop-mediated isothermal amplification (RT-LAMP) assay was developed. The assay was carried out at 58 °C for 40 min, with visual detection of the results using hydroxynaphthol blue dye. The limit of detection of the assay was about 10^2.0^ EID 50/reaction for the M and H5 genes of H5N1 HPAIV [85].

In 2020, an RT-LAMP assay was developed with fluorescence oligonucleotides connected by the backward loop primer for the detection of two subtypes of influenza viruses (influenza A (A/H1 and A/H3) and influenza B). The detection limits for this RT-LAMP assay were 10^3^ copies and 10^2^ copies of RNA for influenza A and influenza B viruses, respectively. This multiplex avian influenza RT-LAMP presented sensitivities of 94.62% and 97.50% for influenza A and influenza B clinical samples, respectively, and specificities of 100% for influenza A, influenza B and clinical samples from apparently healthy chickens. Moreover, this RT-LAMP assay had no cross-reactivity with other respiratory viruses [86]. New LAMP assays are being developed every now and then, with the overall goal of improving the assay and making it less costly.

**Table 1:** LAMP/RT-LAMP assay compared with PCR/RT-PCR.

Feature	PCR/RT-PCR	LAMP/RT-LAMP	References
Amplification	Thermal cycling amplification of RNA or DNA	Isothermal amplification of RNA or DNA	[30,87]
Sensitivity	High	High and comparable to PCR	[30]
Specificity	High with proper primer design	High when primers are properly optimized	[88]
Time	2–3 h	30–60 min	[88,89]
Prone to presence of irrelevant DNA	More prone	Less prone	[30]
Equipment and cost	High cost, as it requires a thermal cycler and a regular supply of reagents	Low cost, as it requires minimal equipment	[90]
Method of detection	Gel electrophoresis or fluorescence	Color change, turbidity or fluorescence	[3,32,88,91]
Suitability for field conditions	Typically used in a laboratory setting	Suitable for field conditions or on-site diagnostic facilities	[89]

## 7. LAMP/RT-LAMP Validation and Testing of Clinical Samples for Poultry Respiratory Pathogens

At present, there are no multicenter or large-scale validation studies of the LAMP assay for poultry viral respiratory pathogen diagnosis. Instead, most of these studies are limited to different geographic areas. For instance, an RT-LAMP assay, which was developed to detect H10 subtype avian influenza viruses, was used to screen 1296 oropharyngeal and cloacal swab samples from healthy chickens, ducks, geese, francolins and pigeons. Of the samples tested, eight were positive using both the RT-LAMP assay and virus isolation techniques [92], suggesting that this RT-LAMP assay can efficiently detect H10 viruses. In another study, where 112 clinical samples were tested for H9 influenza viruses, the RT-LAMP assay results were comparable with virus isolation. However, RT-PCR was less sensitive compared with the RT-LAMP assay [93]. In another study, where RT-LAMP was developed to detect H3 avian influenza viruses, 76 cloacal swab samples were tested using RT-LAMP, RT-PCR and virus isolation. The results suggested that the clinical sensitivity of the RT-LAMP assay was consistent with virus isolation [82].

For IB, a total of 102 RNAs isolated from the field samples were subjected to RT-LAMP. All samples that tested positive for this RT-LAMP were inoculated into embryonated chicken egg and incubated after four passages. Embryo lesions, such as dwarfing of embryos and curled toes, were observed, suggestive of IB [71]. In a different study, infectious bronchitis RT-LAMP test results of 35 samples collected from sick chicken flocks compared favorably with RT-qPCR assay results. This IBV-RT-LAMP assay had 100% sensitivity and 100% specificity compared with the RT-qPCR assay [73]. Moreover, another study developed an mRT-LAMP-LFD assay to simultaneously detect IBV and NDV. This mRT-LAMP-LFD assay was validated using positive IBV (144 tissues and 124 swabs) and NDV (87 tissues and 76 swabs) clinical samples and other negative IB and ND samples (fowl adenovirus (FAdV)-positive livers and AIV-positive lungs). This mRT-LAMP-LFD showed the highest mean detection rates (98.65% for IBV and 97.25% for NDV) for different types of clinical samples when conducting IBV or NDV single detection. This suggests that this assay is a great tool for field diagnosis of IB and ND [72].

Pham et al. also compared the sensitivity of the LAMP assay with nested PCR for the detection of NDV in the organs of infected chickens. This study concluded that the results of both LAMP and PCR assays were the same [77]. In another study, where an NDV LAMP assay was validated using a total of 95 wild strains isolated from various birds (including chicken, duck, quail, peafowl or ostrich) and 7 reference strains (including 3 velogenic viruses, 2 lentogenic viruses and 2 asymptomatic viruses), when the results were compared with PCR, it was observed that the NDV LAMP assay amplification was in agreement with that of PCR [91].

With regard to ILT, Xie et al. developed a LAMP assay for the rapid detection of ILTV. This assay was used to screen tracheal swabs from chickens infected naturally and five ILTV strains and compared with PCR. The results indicated that this ILT LAMP assay was 10 times more sensitive [63]. Another ILT-LAMP assay was designed and used to test 237 samples from chickens with respiratory disease. In this study, it was concluded that this ILT-LAMP assay could effectively identify avian infectious laryngotracheitis from a large number of clinical samples [65].

A validation study of the RT-qLAMP assay used for detection of aMPV using 50 RNA samples from both turkeys and chickens was analyzed. In comparison with qRT-PCR, there was 100% concordance between these two assays [3].

## 8. LAMP/RT-LAMP Assay Combined with Other Assays for Diagnosis of Some Poultry Respiratory Viruses

A loop-mediated isothermal amplification and lateral-flow assay (LAMP-LFA), which is capable of simultaneously detecting influenza A and influenza B, was designed. This influenza A/B multiplex LAMP-LFA showed 98% specificity for non-infected human clinical samples, along with sensitivities of 94.1% and 96.6% for influenza A and B clinical samples, respectively [94]. Considering that this LAMP-LFA assay was developed for testing human clinical samples, it is applicable to poultry viral pathogens as well. Loop-mediated isothermal amplification (LAMP) was combined with an optomagnetic nanoparticle-based readout system to enhance high amplification efficiency. This combination showed high sensitivity and rapid detection of Newcastle disease virus RNA [78]. Also, two novel CRISPR-Cas13a combined multiplex isothermal recombinase amplification (MIRA) assays—a fluorescence-based method (Fluorescent-MIRA-Cas13a) and a lateral-flow strip-based platform (LF-MIRA-Cas13a)—have recently been developed for the detection of aMPV [95].

## 9. The Future of LAMP Assays

**Figure 2:**
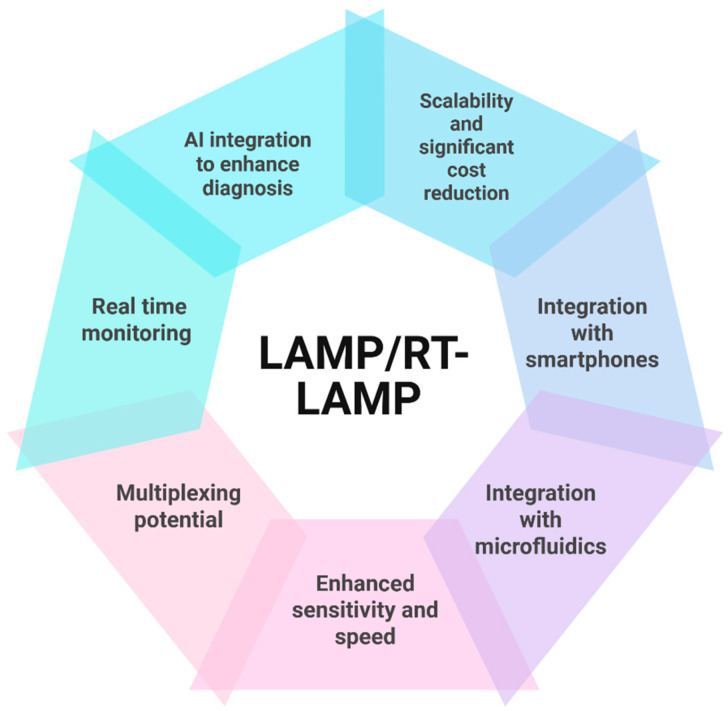
Illustration of recent and possible future advances that make the LAMP/RT-LAMP assay a promising diagnostic tool.

The future of the LAMP assay seems very promising (Figure 2), as there have been a series of advancements aimed at enhancing its development and use, especially in the realm of infectious disease diagnosis in resource-constrained settings. LAMP assays are being integrated with other technologies to enhance disease diagnosis. For instance, LAMP assays have been integrated with microfluidic platforms, providing enhanced automation. LAMP assays also have the potential to be multiplexed, allowing for simultaneous detection of pathogens, enhancing diagnosis in situations of co-infection with two or more pathogens. There is also the possibility of incorporating real-time detection devices, such as sensors, which will allow monitoring of the amplification stage, providing a more quantitative result. Also, artificial intelligence and machine-learning algorithms can be integrated with LAMP, enhancing accurate interpretation of the results. Overall, LAMP assays have potential in global disease surveillance and enhancement of biosecurity, but they require further research for broader adoption in the poultry sector.

## 10. Conclusions

The loop-mediated isothermal amplification assay holds a notable promise for rapid, cost-effective and reliable detection of viral respiratory pathogens in poultry. Its suitability for field conditions, coupled with its speed and ease of use, makes it a reliable tool for disease surveillance and early disease detection on poultry farms. This allows for controlling of poultry viral disease outbreaks early on and eventually improving the biosecurity and overall health management of poultry to boost economic poultry production.
